# Clinical presentation, complications, and outcomes of hospitalized COVID‐19 patients in an academic center with a centralized palliative care consult service

**DOI:** 10.1002/hsr2.423

**Published:** 2021-11-02

**Authors:** Sarah M. Baker, Doug J. Leedy, Jesse Abbott Klafter, Yilin Zhang, Kayla M. Secrest, Tristan R. Osborn, Richard K. Cheng, Seth D. Judson, Susan E. Merel, Carmen Mikacenic, Pavan K. Bhatraju, W. Conrad Liles

**Affiliations:** ^1^ Division of General Internal Medicine, Department of Medicine University of Washington Seattle Washington USA; ^2^ University of Washington School of Medicine Seattle Washington USA; ^3^ Division of Cardiology, Department of Medicine University of Washington Seattle Washington USA; ^4^ Division of Pulmonary, Critical Care and Sleep Medicine, Department of Medicine University of Washington Seattle Washington USA; ^5^ Sepsis Center of Research Excellence‐University of Washington (SCORE‐UW), Department of Medicine, University of Washington Seattle Washington USA; ^6^ Division of Allergy and Infectious Disease, Department of Medicine University of Washington Seattle Washington USA; ^7^ Departments of Global Health, Laboratory Medicine ND Pathology, and Pharmacology University of Washington Seattle Washington USA

**Keywords:** COVID‐19 pandemic, end‐of‐life care, goals of care, palliative medicine, SARS‐CoV‐2 virus

## Abstract

**Background and Aims:**

Palliative care is a critical component of the response of a healthcare system to a pandemic. We present risk factors associated with mortality and highlight an operational palliative care consult service in facilitating early identification of risk factors to guide goal‐concordant care and rational utilization of finite healthcare resources during a pandemic.

**Methods:**

In this case series of 100 consecutive patients hospitalized with COVID‐19, we analyzed clinical data, treatment including palliative care, and outcomes in patients with SARS‐CoV‐2 infection admitted to three hospitals in Seattle, Washington. We compared data between patients who were discharged and non‐survivors.

**Results:**

Age (OR 4.67 [1.43, 15.32] ages 65‐79; OR 3.96 [1.05, 14.89] ages 80‐97), dementia (OR 5.62 [1.60, 19.74]), and transfer from a congregate living facility (OR 5.40 [2.07, 14.07]), as well hypoxemia and tachypnea (OR 7.00 [2.91, 22.41]; OR 2.78 [1.11, 6.97]) were associated with mortality. Forty‐one (41%) patients required intensive care and 22 (22%) invasive mechanical ventilation. Forty‐six (46%) patients were seen by the palliative care service, resulting in a change of resuscitation status in 54% of admitted patients. Fifty‐eight (58%) patients recovered and were discharged, 34 (34%) died, and eight (8%) remained hospitalized, of which seven ultimately survived and one died.

**Conclusions:**

Older age, dementia, and congregate living were associated with mortality. Early discussions of goals of care facilitated by an operational palliative care consult service can effectively guide goal‐concordant care in patients at high risk for mortality during a pandemic. Development of a functional palliative care consult service is an important component of pandemic planning.

## 1 INTRODUCTION

Since the identification of severe acute respiratory syndrome coronavirus 2 (SARS‐CoV‐2) in China in early December 2019, a wave of infection has spread across the world. Considerable variability exists among studies evaluating clinical characteristics and risk factors associated with outcomes.^1^, ^2^, ^3^, ^4^, ^5^, ^6^ Although some variation may be driven by case ascertainment bias, underlying differences in demographics, socioeconomics, access to care, and clinical characteristics across countries likely drive true differences in prevalence of disease and risk for mortality. With the first case in the United States identified in Washington State, the greater Seattle area was the initial epicenter for transmission in the country.^7^, ^8^, ^9^ In this case series, we describe clinical characteristics, interventions including palliative care consultation, and risk factors associated with COVID‐19 among the initial hospitalized cases at three major hospitals in Seattle. We focus on palliative care involvement as a crucial component of the pandemic response, including a description of the effect of our system‐wide Palliative Care Response Plan in the COVID‐19 pandemic, while highlighting the importance of goal‐concordant care.

## 2 METHODS

### 2.1 Study design

Symptomatic, non‐pregnant adult patients (age ≥18 years) with laboratory‐confirmed SARS‐CoV‐2 admitted consecutively three University of Washington (UW) hospitals (UW Medical Center – Montlake, UW Medical Center – Northwest, and Harborview Medical Center) between February 24 and March 30, 2020 were included. This study was approved by the institutional review board of the University of Washington (STUDY00009893). Diagnosis of SARS‐CoV‐2 infection was performed in the clinical virology laboratory of UW by detection of SARS‐CoV‐2 RNA with real‐time PCR. Demographic, clinical, laboratory, and radiological characteristics and COVID‐19‐specific treatment and outcomes data were extracted from electronic medical records by manual chart review.

### 2.2 Patient and public involvement

Patients were not involved in the development of the study design.

### 2.3 Definitions

Patients admitted from congregate living resided at a skilled nursing facility (SNF), a long‐term care facility (LTAC), or an assisted‐living facility (ALF). Timing of symptom onset was estimated from chart review. Fever was defined as a core temperature ≥38°C. Hypoxemia was defined as SaO_2_ <90% on presentation or <88% in patients with chronic lung disease, or new requirement of supplemental O_2_. Tachycardia was defined as a heart rate of ≥100 beats per minute. Tachypnea was defined as a respiratory rate of ≥20 respirations per minute. Immunosuppression was defined as a history of solid organ or bone marrow transplant, immunodeficiency, hematologic malignancy, active chemotherapy, neutropenia (absolute neutrophil count <500 × 10^9^/L), or the use of biologic agents for immunosuppression or corticosteroid equivalent >20 mg/day of prednisone. Malignancy included active cancer, excluding non‐melanoma skin cancers. Cardiovascular disease was defined as hypertension, coronary artery disease, congestive heart failure, or a history of cerebrovascular accident. Dementia was included as a comorbidity in those with a diagnosis of dementia in their medical chart. Leukocytosis was defined as a leukocyte count ≥10.0 × 10^9^/L; neutrophilia as neutrophil count >7.0 × 10^9^/L; and lymphopenia as lymphocyte count <1.0 × 10^9^/L. Acute myocardial injury was defined as troponin‐I above the 99th percentile of the institutional upper reference limit for normal, regardless of new onset abnormalities on electrocardiography or echocardiography. Arrhythmias were sub‐classified as atrial fibrillation, atrial flutter, bradycardia, ventricular tachycardia, and ventricular fibrillation persisting for more than 30 seconds, as documented in the medical record or a 12‐lead electrocardiogram. Cardiomyopathy was defined as newly reduced left ventricular ejection fraction of <50% on transthoracic echocardiography by biplane Simpson's method. The Berlin criteria were used to define acute respiratory distress syndrome (ARDS).^10^ Shock was defined by the use of supportive interventions to maintain arterial blood pressure.

### 2.4 Statistical analysis

Continuous variables were presented as medians and interquartile ranges (IQR), and categorical variables as counts and percentages. Among patients with known outcomes of death or discharge, we compared between‐group differences using Mann–Whitney *U* tests for continuous variables and Pearson's Chi‐squared or Fisher's exact tests for categorical variables. Univariate logistic regression models were used to identify factors associated with intensive care unit (ICU) admission, invasive mechanical ventilation (IMV), and death. For risk of ICU admission and IMV, the entire sample was included in these models since these events tended to occur early in the hospital course. For risk of death, the sample was restricted to patients with a definitive outcome of discharge or death. A two‐sided *P*‐value ≤.05 was considered to be statistically significant. All statistical analyses were performed using STATA 16.1 (Stata Corp.,Texas).

## 3 RESULTS

### 3.1 Demographic and clinical characteristics

Among the 100 consecutive hospitalized patients with COVID‐19, the mean age was 67 years (IQR 54‐78) and 55% were male. Cough (76%), fever (60%), and dyspnea (60%) were the most common presenting symptoms (Figure S1). Of the 100 patients, 85 (85%) had at least one coexisting medical condition, with cardiovascular disease (66%), hypertension (54%), diabetes mellitus (37%), and obesity with BMI ≥35 (31%) being the most common (Table 1).

**TABLE 1:** Patient demographics and clinical data by outcome

	Total N = 100 No. (%)	Remain inpatient N = 8 No. (%)	Discharged N = 58 No. (%)	Died N = 34 No. (%)	*P*‐value^a^
Age group (years)					
20‐49	15 (15.0)	2 (25.0)	10 (17.2)	3 (8.8)	.030
50‐64	29 (29.0)	2 (25.0)	22 (37.9)	5 (14.7)	
65‐79	36 (36.0)	3 (37.5)	16 (27.6)	17 (50.0)	
80‐97	20 (20.0)	1 (12.5)	10 (17.2)	9 (26.5)	
Sex					
Female	45 (45.0)	4 (50.0)	29 (50.0)	12 (35.3)	.17
Male	55 (55.0)	4 (50.0)	29 (50.0)	22 (64.7)	.17
Housing					
Congregate living facility	28 (28.0)	0 (0.0)	10 (17.2)	18 (52.9)	<.001
Comorbidities					
Cardiovascular disease^b^	66 (66.0)	6 (75.0)	37 (63.8)	23 (67.6)	.71
Hypertension	54 (54.0)	6 (75.0)	31 (53.4)	17 (50.0)	.75
CHF	20 (20.0)	1 (12.5)	11 (19.0)	8 (23.5)	.60
CVA	17 (17.0)	2 (25.0)	8 (13.8)	7 (20.6)	.39
CAD	12 (12.0)	0 (0.0)	5 (8.6)	7 (20.6)	.12
Diabetes mellitus	37 (37.0)	3 (37.5)	22 (37.9)	12 (35.3)	.80
Obesity (BMI ≥ 35)	31 (31.0)	2 (25.0)	19 (33.3)	10 (32.3)	.92
Chronic kidney disease	21 (21.0)	0 (0.0)	10 (17.2)	11 (324)	.10
Obstructive lung disease	19 (19.0)	0 (0.0)	10 (17.2)	9 (26.5)	.29
Immunosuppression	18 (18.0)	0 (0.0)	12 (20.7)	6 (17.6)	.72
Dementia	14 (14.0)	0 (0.0)	4 (6.9)	10 (29.4)	.006
Presenting vital sign abnormalities^c^					
Hypoxemia	64 (64.0)	4 (50.0)	30 (51.7)	30 (88.2)	<.001
Tachypnea	59 (59.0)	5 (62.5)	29 (50.0)	25 (73.5)	.027
Fever	33 (33.3)	2 (25.0)	18 (31.6)	13 (38.2)	.52
Tachycardia	32 (32.0)	3 (37.5)	17 (29.3)	12 (35.3)	.55
Oxygen delivery method					
Nasal cannula	79 (79.0)	5 (62.5)	45 (77.6)	29 (85.3)	.37
High‐flow nasal cannula	21 (21.0)	3 (37.5)	10 (17.2)	8 (23.5)	.59
Invasive mechanical ventilation	22 (22.0)	5 (62.5)	7 (12.1)	10 (29.4)	.05
Proning	8 (8.0)	4 (50.0)	2 (3.4)	2 (5.9)	.62
Complications					
ARDS	26 (26.0)	4 (50.0)	8 (13.8)	14 (41.2)	.005
Shock	19 (19.0)	3 (37.5)	5 (8.6)	11 (32.4)	.008
Arrhythmia	18 (18.0)	1 (12.5)	7 (12.1)	10 (29.4)	.05
Cardiomyopathy	3 (3.0)	1 (12.5)	0 (0.00)	2 (5.9)	.13
Hospital unit or service					
Intensive care unit	41 (41.0)	5 (62.5)	19 (32.8)	17 (50.0)	.10
Acute care unit	59 (59.0)	3 (37.5)	39 (67.2)	17 (50.0)	.10

*Note*: Data are presented as N (percent) for categorical variables. Percentages are calculated on the number of patients with that value, and some variables, such as laboratory values, were unavailable for a few patients. The following interventions/medications were omitted from the table due to low frequency of use: NIPPV (3 total), ECMO (3), RRT (4), lopinavir/ritonavir (0), remdesivir compassionate use (3).

^a^ *P*‐Values compare discharged and died groups only; admitted patients were excluded from statistical comparisons. Chi‐squared, Mann–Whitney, and Fisher's exact tests were used as appropriate.

^b^ See text.

^c^ See text.

Abbreviations: ARDS, acute respiratory distress syndrome; BMI, body mass index (kg/m^2^); CAD, coronary artery disease; CHF, congestive heart failure; CVA, cerebrovascular accident; ICU, intensive care unit; IMV, invasive mechanical ventilation.

### 3.2 Hospital admission and length of stay

Fifty‐eight percent of patients recovered to discharge, 8% remained hospitalized, and 34% died. Fifty‐nine percent were treated in the acute care ward alone and 41% in the intensive care unit (ICU) (Table 1). Discharged patients had significantly longer time between symptom onset and admission compared to patients who died (7 days vs 3 days, *P* < .001). The average length of stay was 11 days in discharged patients and 7.5 days in non‐survivors. Each patient was followed through his or her admission or at least 22 days if still hospitalized at time of data censoring.

### 3.3 Comorbidities

**FIGURE 1:**
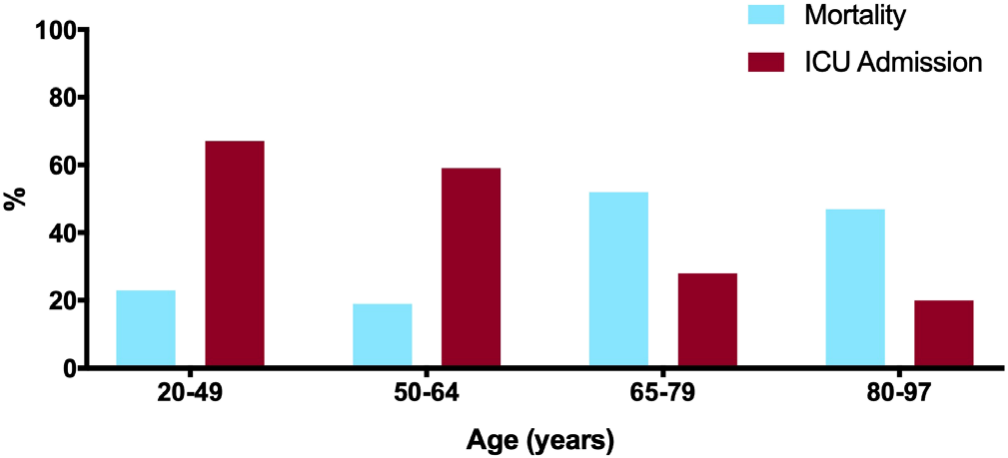
Relationship between mortality and ICU admission by age group. Older patients were more likely to die but were less likely to be admitted to the ICU. (blue = mortality, red = ICU admission)

Older patients were more likely to die than be discharged, with the age groups 65 to 79 years and 80 to 97 years having odds of death of 4.7 [95% CI 1.4, 15.3] and 4.0 [1.1, 14.9], respectively, compared to those 50 to 64 years old (Table 2, Figure 1). Older age was associated with decreased odds of ICU admission, with ORs of 0.27 [0.1, 0.77] and 0.18 [0.05, 0.66], respectively, for age groups 65 to 79 years and 80 to 97 years compared to those 50 to 64 years (Table 2, Figure 1). Dementia was associated with increased morality (OR 5.62 [1.60, 19.74]), and patients with dementia were less likely to be admitted to the ICU (OR 0.20 [0.04, 0.95]) (Table 2). Patients who were admitted from a congregate living facility were significantly less likely to be admitted to the ICU (OR 0.16 [0.05, 0.50]) or be intubated (OR 0.09 [0.01, 0.71]). Congregate living was associated with fivefold increased odds of death (OR 5.40 [2.07, 14.07]).

**TABLE 2:** Odds of intensive care unit (ICU) admission, invasive mechanical ventilation (IMV), and death

	ICU admission	Invasive mechanical ventilation	Death
	OR^a^ [95% CI]	OR [95% CI]	OR [95% CI]
Age (years)			
20‐49	1.41 [0.38, 5.20]	1.31 [0.34, 5.05]	1.32 [0.26, 6.64]
50‐64	Reference	Reference	Reference
65‐79	0.27* [0.10, 0.77]	0.75 [0.24, 2.33]	4.67* [1.43, 15.32]
80‐97	0.18* [0.05, 0.66]	0.14 [0.02, 1.21]	3.96* [1.05, 14.89]
Sex			
Female	Reference	Reference	Reference
Male	1.79 [0.79, 4.05]	2.67 [0.94, 7.53]	1.83 [0.77, 4.38]
Housing			
Congregate living facility	0.16** [0.05, 0.50]	0.09* [0.01, 0.71]	5.40*** [2.07, 14.07]
Comorbidities			
Cardiovascular disease	0.48 [0.20, 1.10]	0.42 [0.16, 1.10]	1.19 [0.48, 2.91]
Diabetes mellitus	1.97 [0.86, 4.50]	1.24 [0.47, 3.25]	0.89 [0.37, 2.15]
Obesity (BMI ≥35)	2.71* [1.12, 6.52]	3.47* [1.29, 9.33]	0.95 [0.37, 2.42]
Chronic kidney disease	0.86 [0.32, 2.30]	0.53 [0.14, 1.98]	2.30 [0.85, 6.18]
Obstructive lung disease	0.21* [0.06, 0.78]	0.61 [0.16, 2.33]	1.73 [0.62, 4.80]
Dementia	0.20* [0.04, 0.95]	1.00 [1.00, 1.00]	5.62** [1.60,19.74]
Presenting vital sign abnormalities^b^			
Hypoxemia	3.73**[1.48, 9.41]	3.13 [0.97, 10.12]	7.00** [2.19, 22.41]
Tachypnea	3.43** [1.43, 8.25]	2.91 [0.98, 8.69]	2.78* [1.11, 6.97]
Fever	1.54 [0.66, 3.59]	0.92 [0.33, 2.52]	1.34 [0.55, 3.26]
Tachycardia	2.08 [0.88,4.88]	1.29 [0.48, 3.47]	1.32 [0.53, 3.24]
Laboratory abnormalities on admission			
Leukocytosis	16.15*** [3.44, 75.88]	17.28*** [5.02, 59.43]	10.48*** [2.69, 40.87]
Neutrophilia	3.68** [1.47, 9.23]	5.87*** [2.12, 16.30]	2.76* [1.08, 7.07]
Lymphopenia	1.00 [0.44, 2.24]	0.81 [0.31, 2.10]	2.00 [0.82, 4.86]
Troponin‐I ≥ 0.04 ng/mL	1.29 [0.43, 3.88]	1.97 [0.59, 6.53]	2.33 [0.71, 7.65]
Elevated liver function tests^c^	1.71 [0.74, 3.96]	2.35 [0.89, 6.18]	0.89 [0.35, 2.25]
Elevated venous lactate^d^	0.83 [0.24, 2.90]	1.48 [0.33, 6.70]	4.93* [1.21, 20.16]

Abbreviations: BMI, body mass index (kg/m^2^); ICU, intensive care unit; IMV, invasive mechanical ventilation.

^a^ Statistical significance: * *P* ≤ .05; ** *P* ≤ .01; ***P ≤ .001 based on univariate logistic regression.

^b^ Hypoxemia, ≤90% on pulse oximetry or newly requiring supplemental oxygen; tachypnea, respiratory rate ≥20 breaths per minute; fever, oral, or temporal temperature ≥38°C; tachycardia, heart rate ≥100 beats per minute.

^c^ Aspartate aminotransferase or alanine aminotransferase greater than upper limit of normal.

^d^ Venous lactate greater than upper limit of normal.

### 3.4 Vital signs and end‐organ dysfunction

Hypoxemia and tachypnea were present in 64% and 59% of patients, respectively (Table 1). Hypoxemia on presentation was associated with increased odds of ICU admission (OR 3.72 [1.48, 9.41]) and death (OR 7.00 [2.19, 22.41]) (Table 2). Tachypnea on presentation was associated with increased odds of ICU admission (OR 3.43 [1.43, 8.25]) and death (OR 2.78 [1.11, 6.97]) (Table 2). Eighty‐six percent of patients required oxygen therapy during admission. Most patients (79%) were placed on nasal cannula, 20% received high‐flow nasal cannula, and 22% required intermittent mandatory ventilation (IMV) (Table 1). Abnormal chest image was common (90%) on presentation, with bilateral patchy opacities (73%) being the most common finding (Table S1).

The most common complications were ARDS (26%), shock (19%), and cardiac arrhythmia (18%) (Table 1). The frequency of complications was significantly higher in patients who died compared to patients discharged. Forty‐one percent of deceased patients developed ARDS compared to 13.8% of discharged patients (*P* = .005). A higher proportion of deceased patients developed shock requiring vasopressor support compared to discharged patients (32.4% vs 8.6%, respectively, *P* = .008). Cardiac arrhythmia was observed in 18% of all patients and was more commonly seen in patients who died (29.4% vs 10.5%, *P* = .052). Atrial fibrillation was the most common arrhythmia (58.8%) (Table 1).

### 3.5 Profile of laboratory abnormalities

Seventeen percent of patients presented with leukocytosis, primarily neutrophil‐predominant, which was associated with significantly increased risk of ICU admission (OR 16.15 [3.44, 75.88]), IMV (OR 17.28 [5.02, 59.43]), and death (OR 10.48 [2.69, 40.87]). Lymphopenia was common on admission (58.6%) but not associated with severe disease or death. Elevated D‐dimer and elevated lactate were significantly associated with death (*P* = .005 and *P* = .024, respectively). Higher peak troponin‐I and brain natriuretic peptide (BNP) levels during hospitalization were significantly associated with death (*P* < .001 and *P* = .004, respectively) (Table S1).

### 3.6 Resuscitation status and palliative care

A formal palliative care consultation was documented in 46% of all patients, 67.6% of those who died, and 31% of those who survived to discharge (Table 3). Similar percentages of patients in the ICU and acute care settings had palliative care consultations. The Palliative Care Response Plan service was most frequently consulted by patients aged 65 to 79 years (69%), followed by patients aged 50 to 64 years (41%). A quarter of patients aged 20 to 49 years and 80 to 97 years had a formal palliative care consultation. Overall, 33% of patients changed their code status during hospitalization, from Full Code to Do Not Resuscitate (DNR)/Do Not Intubate (DNI), or DNR/Intubation OK. Of those seen by a palliative care consultant, 56% changed their code status, and of those who died, 59% had their code status changed prior to death. In contrast, only 16% of patients who survived to discharge changed their code status.

**TABLE 3:** Formal palliative care consultation

	Total N = 100 No. (%)	Remain inpatient N = 8 No. (%)	Discharged N = 58 No. (%)	Died N = 34 No. (%)	*P*‐value
Formal palliative care consult	46 (46.0)	5 (62.5)	18 (31.0)	23 (67.6)	<.001^a^
Code status change^b^	33 (33.0)	4 (50.0)	9 (15.5)	20 (58.8)	<.001^a^
Palliative care consult^c^	25 (75.7)	3 (75.0)	8 (88.9)	14 (70.0)	
No palliative care consult^c^	8 (24.3)	1 (25.0)	1 (11.1)	6 (30.0)	<.001^d^

Formal palliative care consult by age (years)	20‐49 N = 15 No. (%)	50‐64 N = 29 No. (%)	65‐79 N = 36 No. (%)	80‐97 N = 20 No. (%)	
	4 (26.6)	12 (41.4)	25 (69.4)	5 (25.0)	NS

Abbreviation: NS, not significant.

^a^ *P*‐values compare discharged and died groups only. Pearson's Chi‐squared test was used for all comparisons.

^b^ From Full Code to Do Not Resuscitate/Do Not Intubate (DNR/DNI) or DNR/Intubation OK.

^c^ Number and percentage reflects patients who changed code status while hospitalized.

^d^ *P*‐value compares rate of code status change in all patients with and without palliative care consultation.

### 3.7 Medical therapies

Fifty‐two percent of patients were treated with hydroxychloroquine and 51% received azithromycin, typically as part of empirical bacterial community‐acquired pneumonia treatment. Treatment with hydroxychloroquine or azithromycin was not associated with death. There was no association between the incidence of arrhythmia and hydroxychloroquine (data not shown). Seventeen percent of patients received tocilizumab, three patients received remdesivir for compassionate use, and an additional 25 patients were enrolled in a randomized, double‐blinded, placebo‐controlled trial of remdesivir vs placebo with a 1:1 randomization scheme (*NCT04280705*). No medication treatment was associated with either discharge or death (Table S2). No patients received corticosteroids as treatment for COVID‐19 infection during this early phase of the pandemic.

## 4 DISCUSSION

In this study, we report the clinical characteristics, outcomes, and risk factors associated with ICU admission, IMV, and mortality among 100 consecutive patients with documented COVID‐19 admitted to three hospitals in Seattle. The overall in‐hospital mortality rate in these patients was 34%, which is comparable to that in studies of hospitalized patients in Wuhan, China^11^, ^12^ as well as a recent case series from New York City.^13^ Of the 34 patients who died, the mean age was 72% and 64.7% were male. In contrast to other studies, in our study the time from symptom‐onset to admission was significantly shorter among patients who died, while patients requiring the ICU had abrupt deteriorations in respiratory status with subsequent need for IMV, typically 1 day after admission.^2^ Our findings suggest that patients with poor outcomes have a rapid progression of disease, perhaps due to shorter time from symptom onset to hospitalization, or, alternatively, these patients experienced subtle symptoms early on that went unnoticed.

Early case series from China reported fever, cough, and fatigue as the most frequently reported symptoms of COVID‐19 on presentation.^9^, ^10^, ^11^, ^12^ Gastrointestinal (GI) symptoms such as nausea, vomiting, and diarrhea were reported in <5% of patients.^4^ Although fever and cough were common among our patients, nausea or vomiting was present in 24% and diarrhea in 29%. This is consistent with more recent studies suggesting that GI symptoms may be underreported.^14^, ^15^ Importantly, under‐recognition of GI manifestations of COVID‐19 may lead to delayed diagnoses, increasing the spread from undiagnosed individuals.

In our study, dementia was the only comorbidity significantly associated with mortality. With regard to other comorbidities, cardiovascular disease, obesity, and diabetes mellitus were the most common in our patients, which is consistent with a recent report of adult patients with COVID‐19 in the United States.^15^ Obesity was shown to be an independent risk factor for death in H1N1 influenza, and higher BMI has been associated with more severe disease and death in COVID‐19.^16^, ^17^, ^18^ In our study, obesity was a common comorbidity; 47.9% of patients had a BMI ≥30% and 31% of patients had a BMI ≥35 (Table 1). Importantly, BMI ≥35 was associated with an increased risk of both ICU admission and intubation. This association may be due to a higher prevalence of chronic diseases in this population, decreased respiratory reserve, and increased inflammatory cytokines.^16^

Compared to this national cohort, chronic renal disease and immunosuppression were more common among our patients (21% vs 13% and 18% vs 9.6%, respectively). Previous reports from China also reported that hypertension, diabetes, and chronic lung disease were associated with ARDS or death.^11^, ^12^, ^19^

In our study, 40.6% of patients had evidence of myocardial injury during admission. Notably, this is even greater than reported in previous studies, which demonstrated substantial rates of acute myocardial injury in COVID‐19, ranging from 7.2% to 27.8%.^3^, ^12^, ^20^, ^21^, ^22^ Mechanisms for myocardial injury remain unclear, but proposed pathways include demand ischemia, plaque rupture, cytokine release syndrome (CRS), myocarditis, or stress cardiomyopathy.^23^ Regardless of the mechanism, in this study, myocardial injury and elevated BNP were associated with mortality. Arrhythmia during hospitalization was observed in 18% of patients. Of those, 58.8% were atrial fibrillation, 23.5% were bradycardia, and 11.8% were ventricular tachycardia/ventricular fibrillation. The predominance of atrial fibrillation suggests that arrhythmia may be a byproduct of systemic illness and global inflammation.

Specific laboratory abnormalities have begun to emerge as prognostic indicators for SARS‐CoV‐2 infections.^24^, ^25^ Interestingly, leukocytosis, specifically neutrophilia, was associated with ICU admission, IMV, and mortality. These may be signs of dysregulated inflammatory response as observed in sepsis or evidence of a secondary bacterial infection, contributing to death. An elevated D‐dimer was associated with death, which is consistent with the observations of Zhou et al, who reported elevated D‐dimer to be an independent risk factor for mortality.^12^ This may be a marker of inflammation, CRS, venous thromboembolism, and/or disseminated intravascular coagulopathy—all of which have been proposed as underlying mechanisms driving mortality in COVID‐19.^25^, ^26^, ^27^

Consistent with prior reports, older age was associated with increased risk of severe disease and mortality. Age above 65 years was associated with increasing odds of death. Those with dementia or admitted from congregate living facilities experienced increased mortality. Importantly, these findings highlight the significant vulnerability of older cohorts to viral pandemics. Following identification of early outbreaks in local congregate living facilities,^8^ our institution developed a system‐wide Palliative Care Response (PCR) plan in anticipation of these high‐risk patients. This initiative included daily remote palliative care support for the emergency department (ED), ICU, and acute care services.^28^ As such, while palliative care was formally consulted in 46% of cases, clinicians had daily access to palliative care consultants to discuss clinical questions regarding most or all patients. The PCR plan focused on identifying and addressing goals of care, including discussions on code status, in an effort to reduce the risk of unwanted or non‐beneficial cardiopulmonary resuscitation.^28^This plan facilitated early prognosis and goals‐of‐care discussions with patients at risk for poor outcomes and placement of DNR orders when appropriate. Indeed, a higher percentage of patients who were seen by the palliative care team changed their code status during hospitalization. These results align with a study done in an ED, which found that after systematic implementation of palliative care, most patients chose to forgo mechanical ventilation and/or CPR.^29^ Importantly, our data suggest that palliative care interventions resulted in the avoidance of ICU admissions and invasive procedures for many elderly patients with chronic comorbid conditions for whom such aggressive or invasive procedures were not consistent with the goals of care (Figure 1). Consequently, patients 65 years or older were less likely to be admitted to the ICU and very few were intubated. As far as we are aware, this is the first study to describe the impact of systematic implementation of palliative care in the inpatient setting to guide appropriate resource utilization and harmonize clinical trajectory with goals of care.

Our observations regarding the impact of palliative care contrast with those in other countries, such as China and Italy, where the elderly made up a significant portion of critical care admissions. In Italy, 22.8% of patients admitted to intensive care were over the age of 70% and 72% of those patients received IMV.^30^ The strong presence of our palliative care consultation service likely accounts for this difference and was instrumental in ensuring the care delivered aligned with the goals and values of our patients. This is particularly salient as our knowledge of the challenges of extubating patients with COVID‐19 infection evolves. Indeed, more data are emerging on the challenges regarding extubation of COVID‐19 patients.^31^, ^32^ In one study, 88% of those who received mechanical ventilation died.^13^

In the ongoing response to the COVID‐19 pandemic, strategies to ensure access to palliative care consultation for patients and clinicians are essential. As clear risk factors for morbidity and mortality such as those detailed in this study, including age, other medical comorbidities, and laboratory abnormalities, begin to emerge, these data can be used to identify patients who may benefit from early involvement of palliative care. Early involvement may help identify groups not likely to benefit from aggressive care and minimize misalignment of scarce resource allocation in regions that are heavily burdened by the pandemic. This approach to allocation of resources may help to relieve the strain on the system, particularly ICUs, and appropriately avoid medical futility.

This study has several limitations. Similar to any observational study, associations may reflect residual confounding. Complete documentation of presenting symptoms was not obtainable on all patients due to comorbid conditions such as dementia or respiratory distress. Comprehensive laboratory values were not routinely obtained, as laboratory abnormalities associated with COVID‐19 were unknown early on during the pandemic. Because only 3% of patients in our study required veno‐venous extracorporeal membranous oxygenation (ECMO) and only 4% required renal replacement therapy (RRT), we were unable to reliably evaluate the impact of these clinical interventions on outcome. At the time of data censoring, 8 (8%) patients remained hospitalized.

## 5 CONCLUSION

In this case series, 100 consecutive patients admitted to hospital with COVID‐19 in Seattle, presenting clinical characteristics and laboratory indices associated with more severe disease and mortality, were identified. During the initial stages of the COVID‐19 pandemic in Seattle, recognition of risk factors associated with poor prognosis, in conjunction with a systematic palliative care response plan, played an important role in the provision of goal‐concordant care while maintaining functional resilience, rational utilization of finite medical resources, and sustainability of the healthcare system.The provision of an operational PCR plan will be critical for healthcare systems to respond appropriately and effectively to not only the ongoing COVID‐19 pandemic but also other pandemics that will arise inevitably in the future.

## FUNDING

None declared.

## CONFLICT OF INTEREST

The authors declare no conflicts of interest.

## AUTHOR CONTRIBUTION

Conceptualization: Doug J. Leedy, Sarah M. Baker, W. Conrad Liles.

Data Curation: Doug J. Leedy, Kayla M. Secrest, Sarah M. Baker, Tristan R. Osborn, Yilin Zhang, Jesse Abbott Klafter, Seth D. Judson.

Formal Analysis: Jesse Abbott Klafter.

Supervision: W. Conrad Liles.

Writing – Original Draft Preparation: Sarah M. Baker, Doug J. Leedy, Jesse Abbott Klafter, Yilin Zhang, Tristan R. Osborn, Richard K. Cheng, Susan E. Merel, Seth D. Judson.

Writing – Review and Editing: Susan E. Merel, Richard K. Cheng, Pavan K. Bhatraju, W. Conrad Liles, Yilin Zhang, Tristan R. Osborn, Carmen Mikacenic.

All authors have read and approved the final version of the manuscript.

The corresponding author had full access to all of the data in this study and takes complete responsibility for the integrity of the data and the accuracy of the data analysis.

## TRANSPARENCY STATEMENT

W. Conrad Liles affirms that this manuscript is an honest, accurate, and transparent account of the study being reported; that no important aspects of the study have been omitted; and that any discrepancies from the study as planned have been explained.

## ETHICS STATEMENT

This study was approved by the institutional review board of the University of Washington (STUDY00009893).

## Supporting information


**Figure S1.** Symptoms reported by COVID‐19 patients. Among the hospitalized patients with confirmed SARS‐CoV‐2 infection, the percentages of patients reporting particular symptoms at time of admission are shown.

**Table S1.** Laboratory and radiographic findings
**Table S2.** Medical therapies

## Data Availability

The authors confirm that the data supporting the findings of this study are available within the article and its supplementary materials.
