# Supplementary figures and images for: Clinical presentation, complications, and outcomes of hospitalized COVID‐19 patients in an academic center with a centralized palliative care consult service
Source: Health Sci Rep. 2021 Nov 2;4(4):e423. doi: 10.1002/hsr2.423 (PMC8562312; doi:10.1002/hsr2.423)

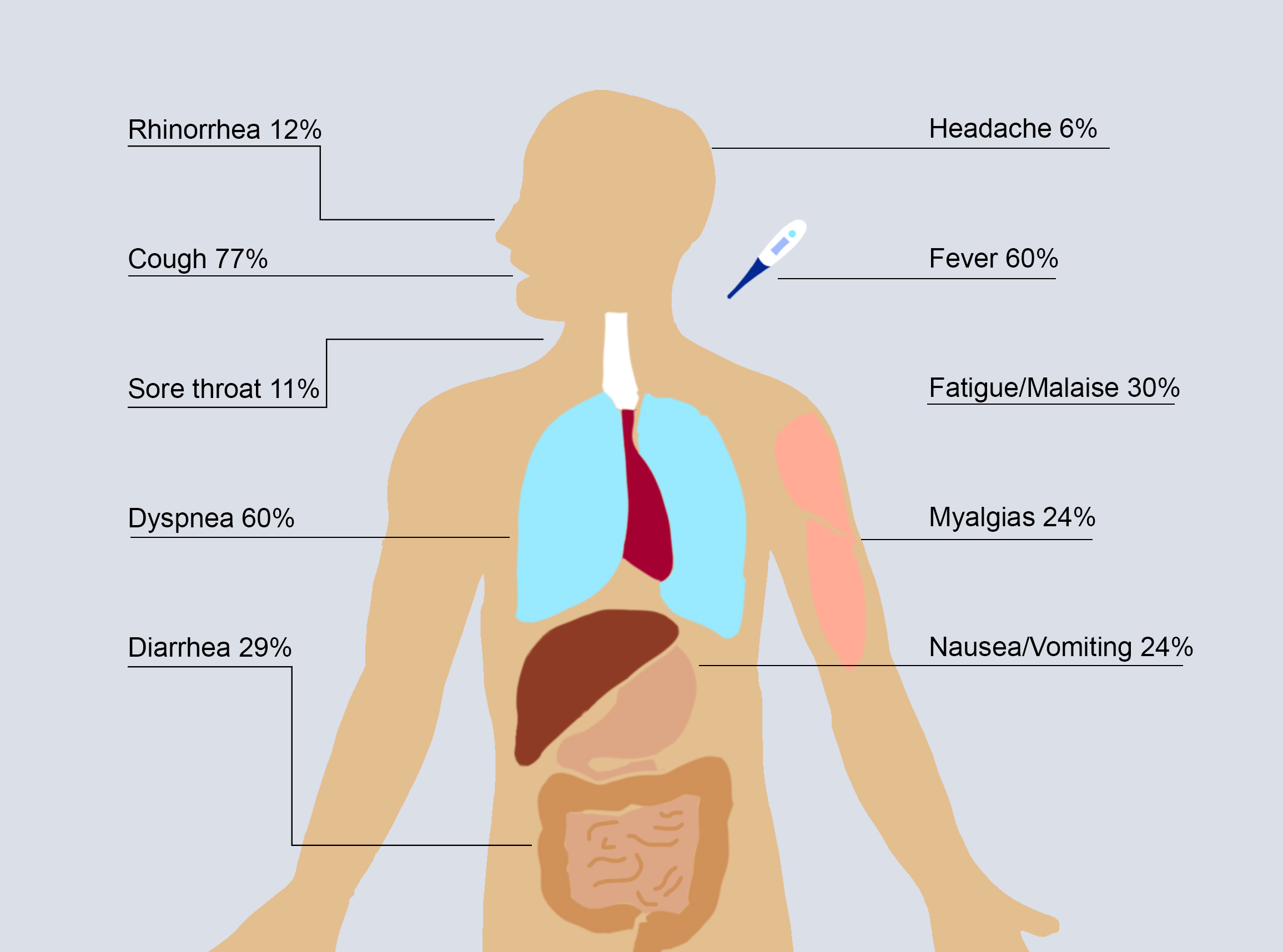

Supplement: Supplementary file 1 — Figure S1. Symptoms reported by COVID‐19 patients. Among the hospitalized patients with confirmed SARS‐CoV‐2 infection, the percentages of patients reporting particular symptoms at time of admission are shown. [file HSR2-4-e423-s002.jpg]
