# Supplementary material for: Clinical presentation, complications, and outcomes of hospitalized COVID‐19 patients in an academic center with a centralized palliative care consult service
Source: Health Sci Rep. 2021 Nov 2;4(4):e423. doi: 10.1002/hsr2.423 (PMC8562312; doi:10.1002/hsr2.423)
Supplement: Supplementary file 2 — Table S1. Laboratory and radiographic findings Table S2. Medical therapies [file HSR2-4-e423-s001.docx]

**Supplemental Data**

**Supplemental Figure S1.** **Symptoms reported by COVID-19 patients.** Among the hospitalized patients with confirmed SARS-CoV-2 infection, the percentages of patients reporting particular symptoms at time of admission are shown.

**Supplemental Table S1. Laboratory and radiographic findings**

|  | | **Total**  **N = 100**  **No. (%)** | **Remain Inpatient**  **N = 8**  **No. (%)** | **Discharged**  **N = 58**  **No. (%)** | **Died**  **N = 34**  **No. (%)** | **p-value^a^** |
| --- | --- | --- | --- | --- | --- | --- |
| **Laboratory Abnormalities**  **on Admission** | |  |  |  |  |  |
|  | Lymphopenia | 58 (58.6) | 7 (87.5) | 29 (50.0) | 22 (66.7) | 0.12 |
|  | Elevated Liver Function Tests^a^ | 34 (34.3) | 5 (62.5) | 19 (32.8) | 10 (30.3) | 0.81 |
|  | Elevated Venous Lactate | 14 (23.7) | 3 (50.0) | 4 (11.4) | 7 (38.9) | 0.031 |
|  | Leukocytosis | 17 (17.2) | 2 (25.0) | 3 (5.2) | 12 (36.4) | <0.001 |
|  |  | **No. (IQR)** | **No. (IQR)** | **No. (IQR)** | **No. (IQR)** | **p-value** |
| **Peak Laboratory Abnormalities**  **During Admission** | |  |  |  |  |  |
|  | IL-6 | 131 (53-589) | 1598 (152-8418) | 79 (57-270) | 131 (45-266) | 0.90 |
|  | Ferritin | 819 (400-1686) | 542 (232-1262) | 733 (400-1414) | 1686 (627-4216) | 0.10 |
|  | D-Dimer | 1.42 (0.90-3.83) | 4.94 (3.47-22.07) | 1.01 (0.58-1.41) | 3.48 (1.46-5.12) | 0.005 |
|  | LDH | 358 (298-459) | 448 (385-692) | 334 (248-382) | 383 (333-695) | 0.05 |
|  | BNP | 155 (51-366) | 179 (77-227) | 107 (27-230) | 318 (136-561) | 0.004 |
|  | Troponin ≥ 0.04ng/ml | 0.00 (0.00-0.05) | 0.05 (0.00-0.40) | 0.00 (0.00-0.00) | 0.04 (0.01-0.13) | <0.001 |
|  | Creatine Kinase | 238 (114-773) | 219 (128-325) | 238 (114-575) | 426 (97-990) | 0.72 |
|  | Venous Lactate | 1.4 (1.1-1.9) | 1.95 (1.7-2.7) | 1.3 (1.1-1.5) | 1.6 (1.2-2.3) | 0.024 |
|  |  | **No. (%)** | **No. (%)** | **No. (%)** | **No. (%)** | **p-value** |
| **Radiographic Findings** | |  |  |  |  |  |
|  | Normal/None/Other | 10 (10.0) | 2 (20.0 | 5 (50.0) | 3 (30.0) |  |
|  | Unilateral Opacity | 17 (17.0) | 1 (5.9) | 12 (70.6) | 4 (23.5) |  |
|  | Bilateral Opacity | 73 (73.0) | 5 (6.8) | 41 (56.2) | 27 (37.0) |  |

^a^Aspartate aminotransferase or alanine aminotransferase > upper limit of normal

Abbreviations: ARB, angiotensin receptor blocker; ACE-I, angiotensin converting enzyme inhibitor; BNP, brain natriuretic peptide; IL-6, interleukin-6; IQR, interquartile range; LDH, lactate dehydrogenase.

**Supplemental Table S2. Medical Therapies**

|  | | **Total**  **N = 100**  **No. (%)** | **Remain Inpatient**  **N = 8**  **No. (%)** | **Discharged**  **N = 58**  **No. (%)** | **Died**  **N = 34**  **No. (%)** | **p-value^a^** |
| --- | --- | --- | --- | --- | --- | --- |
| **Medical Therapies** | |  |  |  |  |  |
|  | Azithromycin | 51 (51.0) | 4 (50.0) | 30 (51.7) | 17 (50.0) | 0.87 |
|  | ACE-I/ARB | 32 (32.0) | 5 (62.5) | 20 (34.5) | 7 (20.6) | 0.16 |
|  | Trial Drug^a^ | 25 (25.0) | 4 (50.0) | 15 (25.9) | 6 (17.6) | 0.45 |
|  | Tocilizumab | 17 (17.0) | 5 (62.5) | 10 (17.2) | 2 (5.9) | 0.20 |
|  | Hydroxychloroquine | 52 (52.0) | 8 (100.0) | 32 (55.2) | 12 (35.3) | 0.070 |

^a^Randomized placebo-controlled trial of remdesivr

The following interventions/medications were omitted from the table due to low frequency of use: lopinavir/ritonavir (0), remdesivir compassionate use (3).
